# Xylol (alle Isomere) – Addendum: Reevaluierung des BAT-Wertes und der Schwangerschaftsgruppe zum BAT-Wert: Beurteilungswerte in biologischem Material

**DOI:** 10.34865/bb133020d10_1ad

**Published:** 2025-03-31

**Authors:** Thomas Jäger, Wobbeke Weistenhöfer, Hans Drexler, Andrea Hartwig, MAK Commission

**Affiliations:** 1 BASF SE. Corporate Health Management Carl-Bosch-Straße 38 67056 Ludwigshafen Deutschland; 2 Friedrich-Alexander-Universität Erlangen-Nürnberg. Institut und Poliklinik für Arbeits-, Sozial- und Umweltmedizin Henkestraße 9–11 91054 Erlangen Deutschland; 3 Institut für Angewandte Biowissenschaften. Abteilung Lebensmittelchemie und Toxikologie. Karlsruher Institut für Technologie (KIT) Adenauerring 20a, Geb. 50.41 76131 Karlsruhe Deutschland; 4 Ständige Senatskommission zur Prüfung gesundheitsschädlicher Arbeitsstoffe. Deutsche Forschungsgemeinschaft, Kennedyallee 40, 53175 Bonn, Deutschland. Weitere Informationen: Ständige Senatskommission zur Prüfung gesundheitsschädlicher Arbeitsstoffe | DFG

**Keywords:** Xylol (alle Isomere), Methylhippursäure, Biologischer Arbeitsstoff-Toleranzwert, BAT-Wert, Entwicklungstoxizität

## Abstract

The German Senate Commission for the Investigation of Health Hazards of Chemical Compounds in the Work Area (MAK Commission) summarised and re-evaluated the data for the biological tolerance value (BAT value) for xylene [1330-20-7] after the occupational exposure limit value (maximum concentration at the workplace, MAK value) for this substance had been lowered from 100 to 50 ml/m^3^ taking into account the increased respiratory volume in the workplace. Since the last evaluation, new publications have been identified from a literature search and relevant studies are described in detail. For the derivation of the BAT value, both occupational field studies and experimental studies showed a good correlation between the xylene concentration in the air and the methylhippuric acid concentration in the urine. Considering the available studies and an increased respiratory volume for experimental studies without physical excercise, an average of 1800 mg methylhippuric acid/g creatinine can be expected after eight-hour exposure to xylene (all isomers) at the level of the MAK value and was therefore established as the BAT value. Sampling should take place at the end of exposure or the end of a shift. In 2021, the MAK value for xylene was re-evaluated with regard to developmental toxicity. No comprehensive studies on developmental neurotoxicity were available, therefore, the assignment of xylene to Pregnancy Risk Group D was confirmed. As the BAT value was derived in correlation to the MAK value, Pregnancy Risk Group D applies to the BAT value as well.

**Table:** 

**BAT-Wert (2024)**	**1800 mg Methylhippursäure/g Kreatinin**
	Probenahmezeitpunkt: Expositionsende bzw. Schichtende
	
**MAK-Wert (2019)**	**50 ml/m^3^ ≙ 220 mg/m^3^**
Spitzenbegrenzung (2001)	Kategorie II, Überschreitungsfaktor 2
Hautresorption (1998)	H
Krebserzeugende Wirkung	–
Fruchtschädigende Wirkung (1988)	Gruppe D

## Reevaluierung

Für Xylol (alle Isomere) wurden im Jahr 1984 Biologische Arbeitsstoff-Toleranzwerte (BAT-Werte) für die Parameter Xylol im Blut und Methylhippursäure im Urin in Korrelation zur maximalen Arbeitsplatzkonzentration (MAK-Wert) (Henschler 1983) aufgestellt und nach Reevaluierung im Jahr 2000 bestätigt (Angerer 1986; Angerer und Krämer 2001). Im Jahr 2015 wurde der BAT-Wert für Xylol im Blut aufgrund der schwer zu realisierenden Probenahme (sehr rasche Elimination von Xylol aus dem Kompartiment Blut und damit notwendige Probenahme möglichst unmittelbar nach Expositionsende) ausgesetzt (Jäger et al. 2016). 

Seit dem Jahr 2016 berücksichtigt die Kommission bei Stoffen, deren MAK-Wert auf systemischen Effekten basiert und aus inhalativen Tierversuchen oder Probandenstudien in Ruhe abgeleitet wurde, das im Vergleich zu den experimentellen Bedingungen erhöhte Atemvolumen am Arbeitsplatz (Hartwig und MAK Commission 2017). Da die Xylolkonzentration im Blut bei dem MAK-Wert in Höhe von 100 ml Xylol/m^3^ bei Berücksichtigung des erhöhten Atemvolumens am Arbeitsplatz über der von MacDonald et al. (2002) abgeleiteten Schwellenkonzentration für pränarkotische Wirkungen von 2 mg/l liegt, wurde der MAK-Wert auf 50 ml Xylol/m^3^ abgesenkt (Hartwig und MAK Commission 2020). Da der BAT-Wert in Höhe von 2000 mg Methylhippursäure (alle Isomere)/l Urin (Angerer 1986) in Korrelation zum damaligen MAK-Wert in Höhe von 100 ml/m^3^ abgeleitet wurde, muss er reevaluiert werden. Zudem wurden seit der letzten Evaluierung des BAT-Wertes neue experimentelle Probandenstudien (Ernstgård et al. 2003; Loizou et al. 1999) und arbeitsmedizinische Feldstudien (Jacobson und McLean 2003; Jang et al. 2001; Rajan et al. 2019) publiziert. 

### Experimentelle Probandenstudien

Loizou et al. (1999) untersuchten in einer toxikokinetischen Studie 4 Personen (2 Männer, 2 Frauen), die einer *m*-Xylolkonzentration von 50 ml/m^3^ ohne körperliche Belastung über 2 × 12 Stunden ausgesetzt waren (jeweils zwei halbstündige expositionsfreie Zeiten nach 4 und 8 Stunden). Die Xylolkonzentration im Blut betrug nach einer Stunde 0,35 mg/l und nach acht Stunden 0,6 mg/l. Die Methylhippursäurekonzentration lag nach 8 Stunden bei 1100 mg/g Kreatinin. 

Ernstgård et al. (2003) untersuchten in einer experimentellen Probandenstudie 17 Personen (9 Frauen, 8 Männer), die unter leichter körperlicher Aktivität (50 Watt) 2 Stunden lang gegen 45 ml *m*-Xylol/m^3^ exponiert waren. Die *m*-Xylolkonzentration im Blut am Expositionsende betrug bei den Frauen 1,23 mg/l und bei den Männern 1,17 mg/l. Die Methylhippursäureausscheidung innerhalb von 24 Stunden nach Exposition betrug 201 mg bei den Frauen und 240 mg bei den Männern. 

Tardif et al. (1991) untersuchten Personen, die ohne körperliche Belastung Xylol (alle Isomere) ausgesetzt waren. Nach Xylol-Expositionen in Höhe von 40 ml/m^3^ (5 Personen, 7 Stunden/Tag, einmal pro Woche, 3 Expositionswochen) oder 80 ml/m^3^ (3 Personen, 4 Stunden an einem Tag) wurden Methylhippursäurekonzentrationen im Urin von 900 mg/g Kreatinin bzw. 810 mg/g Kreatinin bestimmt, wobei die Probenahme während der letzten 4 Stunden der Exposition erfolgte.

Sedivec und Flek (1976) exponierten vier männliche Probanden (Atemminutenvolumen 9 l/min) für 8 Stunden gegen 50 ml Xylol (alle Isomere)/m^3^. In den letzten beiden Stunden der Exposition lagen die Methylhippursäurekonzentrationen im Urin im Bereich von 650,7 bis 1211,3 mg/g Kreatinin (527,4–2259,1 mg/l).

Jang et al. (1997) untersuchten 6 Asiaten und 6 Europäer, die ohne körperliche Belastung 6 Stunden gegen 100 ml *m*-Xylol/m^3^ exponiert waren. Die mittlere *m*-Methylhippursäureausscheidung im Urin war bei den Europäern (2670 mg/g Kreatinin) statistisch signifikant höher als bei den Asiaten (1860 mg/g Kreatinin). 

Ogata et al. (1970) exponierten 23 männliche asiatische Probanden 7 Stunden gegen 100 oder 200 ml *m*-Xylol/m^3^ in Ruhe (Atemminutenvolumen 8,6 l). Die mittlere *m*‑Methylhippursäurekonzentration in Proben, die während der letzten vier Stunden der Exposition gesammelt wurden, betrug 3140 mg/l bzw. 5790 mg/l Urin. 

Eine Übersicht der Ergebnisse aus den experimentellen Probandenstudien gibt Tabelle 1.

**Tab. 1:** Übersicht der experimentellen Probandenstudien mit Exposition gegen Xylol

Studiendesign	Xylole in der Luft [ml/m^3^]	Xylole im Blut [µg/l]	MHA im Urin [mg/g Kreatinin]	Verwendete Faktoren	50-ml/m^3^-Äquivalent^a)^ [mg/g Kreatinin]	Literatur
23 ♂ Asiaten; *m*‐Xylol, 7 h	100	–	3140 mg/l (während Exposition (4–8 h))	EZ: 8/7 EH: 0,5/0,25 KA: 2 KB: 0,71	2548	Ogata et al. 1970
	200	–	5790 mg/l (während Exposition (4–8 h))		2349	
4 ♂ Personen; *o*‐, *m*‐, *p*‐Xylol, 8 h Atemzeitvolumen 9 l/min	50	–	650,7–1211,3 (während der letzten 2 h der Exposition)	KA: 2	1301–2423	Šedivec und Flek 1976
5 ♂ Europäer; Nichtraucher Xylol (alle Isomere), 7 h/d an 3 Tagen (in Woche 1, 3 und 5) ohne körperliche Belastung	40	676 (während (6,5 h) Exposition); 316 (nach Exposition)	900 (während Exposition (3–7 h))	EZ: 8/7 EH: 1,25 KA: 2	2571	Tardif et al. 1991
3 ♂ Europäer; Nichtraucher Xylol (alle Isomere), 4 h, 1 Tag ohne körperliche Belastung	80	1140 (während (3,5 h) Exposition); 479 (nach Exposition)	810 (während Exposition (0–4 h))	EZ: 8/4 EH: 5/8 KA: 2	2025	
6 ♂ Asiaten, 6 ♂ Europäer; *m*‐Xylol, 6 h ohne körperliche Belastung	100	Asiaten: 570 ± 260 (0,5 h nach Exposition)	Asiaten: 1860 ± 740	EZ: 8/6 EH: 0,5 KA: 2	Asiaten: 2480	Jang et al. 1997
		Europäer: 610 ± 170 (0,5 h nach Exposition)	Europäer: 2670 ± 570		Europäer: 3560	
4 Personen (2 ♂; 2 ♀); *m*-Xylol, 2 × 12 h mit 12 h Pause (zusätzlich 0,5 h Pause nach 4 und 8 h)	50 (51 ± 3)	600 (Probenahme nach 8 h Exposition)	≈ 1100 (Probenahme nach 8 h Exposition)	KA: 2	2200	Loizou et al. 1999
17 Personen (9 ♀; 8 ♂); *m*-Xylol, 2 h körperliche Arbeit (50 W)	45	1200 (nach Exposition)	212 mg/24 h	–	–	Ernstgård et al. 2003

^a)^ für 8-stündige Exposition bei körperlicher Aktivität. Einflussfaktoren wie fehlende körperliche Betätigung, fehlender Kreatininbezug und kürzere Expositionszeiten wurden mittels Faktoren berücksichtigt.

EH: Expositionshöhe; EZ: Expositionszeit; h: Stunde; KA: Körperliche Aktivität; KB: Kreatininbezug; MHA: Methylhippursäure

### Arbeitsmedizinische Feldstudien

Eine ausführliche Darstellung der bis zum Jahr 2000 publizierten arbeitsmedizinischen Feldstudien, in denen Personen mit beruflicher Xylol-Belastung untersucht wurden, findet sich bei Angerer und Krämer (2001).

Jang et al. (2001) untersuchten 20 männliche Arbeiter in der Metallverarbeitung mit einer Exposition gegen Ethylbenzol und Xylol. Personenbezogene Luftmessungen wurden arbeitstäglich eine Woche lang durchgeführt und am Ende der Schicht eine Urinprobe gewonnen. Die mittlere Xylolbelastung in der Luft betrug 12,77 ml/m^3^ (Bereich: 2,5–61,6 ml/m^3^). Die Untersuchung zeigte eine statistisch signifikante Korrelation zwischen der Xylolkonzentration in der Luft und der Konzentration der Methylhippursäure (alle Isomeren) im Urin (r^2^ = 0,503). Einer äußeren Belastung in Höhe des MAK-Wertes von 50 ml/m^3^ entsprach in dieser Studie eine Methylhippursäurekonzentration im Urin von 1011 mg/g Kreatinin. In der gleichen Arbeit wurde ein physiologisch basiertes pharmakokinetisches (PBPK)-Modell verwendet, um den Einfluss einer Koexposition von Xylol und Ethylbenzol zu berücksichtigen. Bei einer Exposition gegen 100 ml Xylol/m^3^ und gleichzeitiger Exposition gegen 25 ml Ethylbenzol/m^3^ errechnete das Modell eine Methylhippursäurekonzentration von 1550 mg/g Kreatinin.

Jacobson und McLean (2003) untersuchten 20 Personen (3 Lackierer, 11 Drucker, 2 Maler, 3 Autolackierer, 1 Labormitarbeiter) mit beruflicher Xylolbelastung. Die mittlere Xylolkonzentration in der Luft betrug 3,4 ± 3,6 ml/m^3^ (Bereich: 0,0–14,4 ml/m^3^). Die Methylhippursäurekonzentration wurde in Urinproben vor und nach der Schicht bestimmt. Es bestand eine Korrelation zwischen der Gesamtxylolkonzentration in der Luft und dem kreatininbezogenen Methylhippursäuregehalt (alle Isomeren) im Urin der Beschäftigten (r^2^ = 0,579). Ähnliche Ergebnisse ergaben sich bei der Betrachtung der einzelnen Isomere. Eine äußere Belastung in Höhe des MAK-Werts von 50 ml Xylol/m^3^ würde eine Methylhippursäurekonzentration im Urin von 735 mg/g Kreatinin entsprechen.

Rajan et al. (2019) untersuchten 45 Urinproben von Personen mit unterschiedlicher beruflicher Exposition gegen Xylol. Gruppe 1 bestand aus 5 Medizintechnikern mit einer 50-Stunden-Woche, Gruppe 2 aus Medizintechnikern mit einer 30-Stunden-Woche, Gruppe 3 aus 15 Malern und Gruppe 4 war ein Kontrollkollektiv ohne berufliche Xylolbelastung. Die mittlere *o*-, *m*-Methylhippursäurekonzentration im Urin betrug in Gruppe 1 240,0 ± 54,77 mg/g Kreatinin, in Gruppe 2 33,0 ± 8,23 mg/g Kreatinin, in Gruppe 3 25,3 ± 13,02 mg/g Kreatinin und in der Vergleichsgruppe 4 0,2 ± 0,12 mg/g Kreatinin. Mit Hilfe eines Fragebogens wurde der Gesundheitszustand der Studienteilnehmer erfasst. Die Studienteilnehmer beschrieben das Auftreten von Augen-, Nasen- und Rachenreizungen, Schwindel, Schläfrigkeit, Bauchschmerzen, Appetitlosigkeit, unsicherem Gang und Dermatitis. Diese Studie ist die einzige, in der nach Exposition gegen Xylol bei solchen niedrigen Methylhippursäurekonzentrationen Symptome berichtet werden. Da in dieser Studie keine Luftmessungen publiziert wurden, kann diese Studie nicht zur Ableitung eines BAT-Wertes in Korrelation zum MAK-Wert in Höhe von 50 ml Xylol/m^3^ herangezogen werden.

Tabelle 2 gibt eine Übersicht über die Ergebnisse der arbeitsmedizinischen Feldstudien mit Exposition gegen Xylol und den daraus berechneten MAK-Wert-Äquivalenten.

**Tab. 2:** Übersicht der Feldstudien mit Exposition gegen Xylol

Exponierte/Tätigkeit	Xylole in der Luft [ml/m^3^]^a)^	Xylole im Blut [µg/l]	Berechnetes 50-ml-Xylol/m^3^-Äquivalent [µg Xylol/l]	*o*-, *m*-, *p*-MHA im Urin [mg/g Kreatinin]	Gleichung	Berechnetes 50-ml-Xylol/m^3^-Äquivalent (*o*-, *m*-, *p*-MHA)	Literatur
15 Maler (14 ♂, 1 ♀); *o*‐, *m*‐, *p*‐Xylol, Ethylbenzol	Bereich: 3–70		–				Engström et al. 1978
	≤ 20	0,07–0,42					
	21–40	0,33–0,62					
	> 40	0,31–1,36					
	50		–	665 (nur *m*-, *p*-MHA)	nicht angegeben	764 mg/g Kreatinin (*o*-, *m*-, *p*-MHA)	
35 ♂ Lackierer (6 unterschiedliche Arbeitsplätze); o‐, m‐, p‐Xylol, Ethylbenzol	MW: 12,8 4,6–19,4^b)^	MW: 164 58–226^b)^	641	MW: 411 mg/l 286–588 mg/l^b)^	nicht angegeben	≈ 1000 mg/l (≙ 715 mg/g Kreatinin)	Angerer und Wulf 1985
14 Arbeiter (Entfetten von Metallteilen)	MW: 75 Bereich: 25–112	MW: 1370 Bereich: 340–3100 y = 0,0189x – 0,0863; r = 0,66	859	MW: 1195 mg/l Bereich: 501–2458 mg/l	y = 14,43x – 82,66; r = 0,73	639 mg/l (≙ 456 mg/g Kreatinin)	Zinser et al. 1985
121 Lackierer (♂)^c)^	MW: 3,8 Max: 61	nicht untersucht	–	MW: 58 Max: 1201	y = 17,8x +3,8; r = 0,80	894 mg/l (≙ 639 mg/g Kreatinin)	Kawai et al. 1991
51 Lackierer (♂)^c)^	MW: 8 Max: 27	y = 11x – 19; r = 0,88	531	–	y = 15,6x + 49; r = 0,72	829 mg/l (≙ 592 mg/g Kreatinin)	Kawai et al. 1992
175 (107 ♂, 68 ♀), Drucker, Kunststoffherstellung^c)^	MW: 14 Max: 175	nicht untersucht	–	–	y = 13x + 31; r = 0,82	681 mg/l (≙ 568 mg/g Kreatinin)	Inoue et al. 1993
233 (122 ♂, 111 ♀), Drucker, Lackierer, Kunststoffverarbeitung^c)^	MW: 3 Max: 103	nicht untersucht	–	–	y = 14,4x + 37	757 mg/l (≙ 630 mg/g Kreatinin)	Huang et al. 1994
38 Arbeiter^c)^	MW: 14	nicht untersucht	–	320	y = 20x + 0; r = 0,96	1000 mg/l (≙ 714 mg/g Kreatinin)	Ogata et al. 1995
13 ♂ Arbeiter in der Lackherstellung	MW: 29 Bereich: 5–58	MW: 380 Bereich: 63–715 y = 7,5x + 164; r = 0,60	539	MW: 1221 mg/l Bereich: 194–2333 mg/l	y = 32,5x + 290; r = 0,78	1915 mg/l (≙ 1367 mg/g Kreatinin)	Krämer et al. 1999
10 ♂ Lackierer	MW: 8 Bereich: 3–21	MW: 120 Bereich: 24–308 y = 15x + 4; r = 0,75	754	MW: 450 mg/l Bereich: 65–1633 mg/l	y = 73x – 116; r = 0,87	3534 mg/l (≙ 2525 mg/g Kreatinin)	
20 ♂ Arbeiter, Metallverarbeitung^c)^	MW: 12,77 Bereich: 2,5–61,6	nicht untersucht	–	–	y = 19x + 61; r = 0,71	1011 mg/g Kreatinin	Jang et al. 2001
20 (18 ♂, 2 ♀), Lackierer, Maler, Drucker, Autolackierer, Labormitarbeiter	MW: 3,36 ± 3,63 Bereich: 0,03–14,44	nicht untersucht	–	MW: 57,2 ± 60,7 Bereich: 0,2–248,9	y = 12,733x + 14,439; r^2^ = 0,579	651 mg/g Kreatinin	Jacobson und McLean 2003

^a)^ personenbezogen, 8 Stunden

^b)^ Mittelwerte an 6 unterschiedlichen Arbeitsplätzen

^c)^ asiatisches Kollektiv

Max: Maximum; MHA: Methylhippursäure; MW: Mittelwert

## Reevaluierung des BAT-Wertes

Für die Reevaluierung des BAT-Wertes von Xylol stehen sowohl arbeitsmedizinische Feldstudien als auch experimentelle Probandenstudien zur Verfügung. 

Dabei ist zu beachten, dass die Daten von asiatischen Studienkollektiven aufgrund einer abweichenden Enzymausstattung und einer im Vergleich zu Europäern daraus resultierenden geringeren Methylhippursäureausscheidung im Urin nur bedingt zur Evaluation eines BAT-Wertes herangezogen werden können (Angerer und Krämer 2001; Jang et al. 1997).

Aus den Daten der europäischen arbeitsmedizinischen Feldstudien (siehe Tabelle 2) lassen sich zur Exposition in Höhe des MAK-Wertes von 50 ml/m^3^ Äquivalente der Methylhippursäure von im Mittel etwa 1093 mg/g Kreatinin (Bereich: 456–2525 mg/g Kreatinin) berechnen. Die mittlere Xylolkonzentration in der Luft lag in den meisten arbeitsmedizinischen Feldstudien jedoch deutlich unterhalb von 50 ml/m^3^, so dass eine Extrapolation in den höheren Konzentrationsbereich mit gewissen Unsicherheiten verbunden ist. Zudem dürfte eine Koexposition mit Ethylbenzol, wie sie bei vielen Arbeitsplätzen durch den Einsatz von technischem Xylol bzw. C8-Aromaten-Mischungen üblich ist, zu einer verringerten Methylhippursäureausscheidung führen (Engström et al. 1984). In den Berichten der arbeitsmedizinischen Feldstudien fehlen leider Angaben, die eine Abschätzung der physischen Aktivität der Beschäftigten während der Exposition erlauben. 

Aufgrund der definierten Expositionssituation meist in Höhe des aktuellen MAK-Wertes und dem Ausschließen einer dermalen Aufnahme durch direkten Hautkontakt eignen sich experimentelle Probandenstudien zur Ableitung des BAT-Wertes für Xylol. Dabei wurden Studien mit sehr kurzer Expositionsdauer (< 4 h) und/oder mit Konzentrationsangaben von Methylhippursäure in mg/24 h (Ernstgård et al. 2003) bei der Ableitung nicht berücksichtigt. Aus den sehr gut übereinstimmenden Daten der experimentellen Probandenstudien von Tardif et al. (1991), Jang et al. (1997) und Loizou et al. (1999) (siehe Tabelle 1) ergibt sich für eine achtstündige Exposition in Höhe des MAK-Wertes von 50 ml/m^3^ ein mittleres Korrelat für die Ausscheidung der Methylhippursäure am Schichtende von 1295 mg/g Kreatinin (Bereich: 1013–1780 mg/g). Dieser Wert bzw. Wertebereich stimmt dabei recht gut mit dem aus den arbeitsmedizinischen Feldstudien ermittelten Wert und Wertebereich überein.

Der Großteil der experimentellen Probandenstudien zu Xylol wurde jedoch ohne körperliche Betätigung durchgeführt. Entsprechend dem Standardvorgehen der Kommission zur Berücksichtigung des erhöhten Atemminutenvolumens am Arbeitsplatz wird bei Studien ohne körperliche Aktivität eine Korrektur mit dem Faktor 2 vorgenommen (Greim 1998 a; Hartwig und MAK Commission 2017). Gamberale et al. (1978) zeigten in ihrer Studie zum Einfluss der körperlichen Aktivität auf die Xylolaufnahme, dass durch eine Fahrradergometer-Belastung während der halben Expositionszeit die Aufnahme von Xylol während der gesamten Expositionszeit ungefähr doppelt so hoch war wie in Ruhe. Die Methylhippursäurekonzentration im Urin wurde in dieser Studie jedoch nicht untersucht. Die Studie von Ernstgård et al. (2003) zeigt ebenfalls sehr gut den Einfluss der körperlichen Aktivität auf die Xylolkonzentration im Blut. Trotz kürzerer Expositionsdauer wurden mit 1,2 mg Xylol/l Blut doppelt so hohe Werte beobachtet wie in den Studien ohne körperliche Belastung von Tardif et al. (1991), Jang et.al (2001) und Loizou et al. (1999) (siehe Tabelle 1). Aufgrund der sehr kurzen Expositionsdauer und der Angabe der Mehtylhippursäurekonzentration in mg/24 h ist diese Studie für die Ableitung eines BAT-Wertes allerdings nicht geeignet.

Da die hier zugrundeliegenden experimentellen Daten ohne körperliche Betätigung erhoben wurden, ergibt sich bei Anwendung des Faktors 2 für die Berücksichtigung eines erhöhten Atemminutenvolumens am Arbeitsplatz aus den experimentellen Probandenstudien für eine achtstündige Exposition in Höhe des MAK-Wertes von 50 ml/m^3^ ein mittleres Äquivalent der Methylhippursäureausscheidung am Schichtende von 2590 mg/g Kreatinin.

Allerdings deutet die gute Übereinstimmung der Ergebnisse aus den arbeitsmedizinischen Feldstudien und den experimentellen Studien ohne körperliche Belastung darauf hin, dass die körperliche Belastung an den Arbeitsplätzen mit Xylol-Exposition zu keiner relevanten Erhöhung des Atemvolumens geführt hat, und der Standardfaktor von 2 in diesem Fall die tatsächliche Arbeitsleistung an den untersuchten Arbeitsplätzen überschätzt. Darüber hinaus ist bei einer in der Praxis regelhaft auftretenden Koexposition gegen Ethylbenzol eine um etwa 20 % verringerte Methylhippursäureausscheidung zu erwarten (Engström et al. 1984).

In Kenntnis dieser Limitierungen wird in der Zusammenschau aller vorliegenden Feld- und experimentellen Studien als gerundeter Mittelwert 

**ein BAT-Wert von 1800 mg Methylhippursäure (alle Isomere)/g Kreatinin**

abgeleitet.

Die Probenahme erfolgt am Expositionsende bzw. Schichtende.

## BAT-Wert und Schwangerschaft 

Im Jahr 2020 wurde der MAK-Wert von Xylol hinsichtlich der fruchtschädigenden Wirkung reevaluiert (Hartwig und MAK Commission 2021). Kritische Effekte von Xylol sind die akuten neurotoxischen Wirkungen beim Menschen und in tierexperimentellen Studien. Aufgrund der Mischexpositionen und der eingeschränkten Validität sind Humanstudien nicht zur Bewertung der entwicklungsschädigenden Wirkung von Xylol geeignet. Xylol hat sich im Tierversuch als nicht teratogen erwiesen. Die NOAEC (no observed adverse effect concentration) für erniedrigtes fetales Körpergewicht liegt bei Ratten bei 100 ml/m^3^ für *o*-Xylol und technisches Xylol. Die anderen Isomere haben erst bei höheren Konzentrationen diesen Effekt zur Folge. Zudem kommt es vermehrt zu skelettalen Variationen mit *o*-Xylol ab 1000 ml/m^3^ sowie mit *m*- und *p*-Xylol bei 2000 ml/m^3^. Die NOAEC für Maternaltoxizität in Form einer verzögerten Körpergewichtsentwicklung und reduzierter Futteraufnahme beträgt für alle Isomere 100 ml/m^3^ und für das technische Xylol 500 ml/m^3^ (Greim 2004; Saillenfait et al. 2003). Zur Beurteilung der Entwicklungsneurotoxizität liegt nur eine Studie an pränatal exponierten Sprague-Dawley-Ratten vor, bei denen bis zur höchsten Konzentration von 1589 ml *p*‑Xylol/m^3^ keine Effekte bei der Testung der akustischen Schreckreaktion und der lokomotorischen Aktivität in der Postnatalzeit gefunden wurden (Greim 1998 b; Rosen et al. 1986). Da sich jedoch das *o*‑Isomer hinsichtlich der erniedrigten Körpergewichte potenter als das *p*-Isomer gezeigt hat (Saillenfait et al. 2003), das *o*-Isomer in der Studie von Rosen et al. (1986) nicht untersucht worden ist und für die verschiedenen Isomere keine umfassenden Studien zur Entwicklungsneurotoxizität vorliegen, wird die Zuordnung von Xylol zu Schwangerschaftsgruppe D bestätigt. Da der BAT-Wert in Korrelation zum MAK-Wert abgeleitet wurde, gilt für den BAT-Wert ebenfalls Schwangerschaftsgruppe D.

## Interpretation

In den vorangegangenen Begründungen wurde bisher auf einen Kreatininbezug des BAT-Wertes für Methylhippursäure verzichtet. Mehrere Arbeiten haben gezeigt, dass der Bezug der Methylhippursäurekonzentration auf den Kreatiningehalt der Probe zu einer besseren Korrelation der Studienergebnisse führt (Engström et al. 1978; Šedivec und Flek 1976). Darüber hinaus sind die Konzentrationsangaben der Arbeiten, die für die Ableitung des BAT-Wertes herangezogen wurden, überwiegend mit Kreatininbezug. Für die Umrechnung von volumenbezogenen Methylhippursäurekonzentrationen wurde bei Kollektiven mit ausgeglichener Geschlechterverteilung ein Faktor von 0,83 und bei männerdominierten Kollektiven ein Korrekturfaktor von 0,71 verwendet (Bader et al. 2020).
